# Physiologically-Based Pharmacokinetic Modeling for Drug-Drug Interactions of Procainamide and *N*-Acetylprocainamide with Cimetidine, an Inhibitor of rOCT2 and rMATE1, in Rats

**DOI:** 10.3390/pharmaceutics11030108

**Published:** 2019-03-06

**Authors:** Yoo-Seong Jeong, Anusha Balla, Kwang-Hoon Chun, Suk-Jae Chung, Han-Joo Maeng

**Affiliations:** 1 College of Pharmacy, Seoul National University, Seoul 08826, Korea; jus2401@snu.ac.kr; 2 College of Pharmacy, Gachon University, Incheon 21936, Korea; aanushaballa@gmail.com (A.B.); khchun@gachon.ac.kr (K.-H.C.)

**Keywords:** procainamide, *N*-acetylprocainamide, rat organic cation transporter 2, rat multidrug and toxin extrusion protein 1, cimetidine, physiologically-based pharmacokinetic modeling for drug-drug interactions

## Abstract

Previous observations demonstrated that cimetidine decreased the clearance of procainamide (PA) and/or *N*-acetylprocainamide (NAPA; the primary metabolite of PA) resulting in the increased systemic exposure and the decrease of urinary excretion. Despite an abundance of in vitro and in vivo data regarding pharmacokinetic interactions between PA/NAPA and cimetidine, however, a mechanistic approach to elucidate these interactions has not been reported yet. The primary objective of this study was to construct a physiological model that describes pharmacokinetic interactions between PA/NAPA and cimetidine, an inhibitor of rat organic cation transporter 2 (rOCT2) and rat multidrug and toxin extrusion proteins (rMATE1), by performing extensive in vivo and in vitro pharmacokinetic studies for PA and NAPA performed in the absence or presence of cimetidine in rats. When a single intravenous injection of PA HCl (10 mg/kg) was administered to rats, co-administration of cimetidine (100 mg/kg) significantly increased systemic exposure and decreased the systemic (*CL*) and renal (*CL*_R_) clearance of PA, and reduced its tissue distribution. Similarly, cimetidine significantly decreased the *CL*_R_ of NAPA formed by the metabolism of PA and increased the AUC of NAPA. Considering that these drugs could share similar renal secretory pathways (e.g., via rOCT2 and rMATE1), a physiologically-based pharmacokinetic (PBPK) model incorporating semi-mechanistic kidney compartments was devised to predict drug-drug interactions (DDIs). Using our proposed PBPK model, DDIs between PA/NAPA and cimetidine were successfully predicted for the plasma concentrations and urinary excretion profiles of PA and NAPA observed in rats. Moreover, sensitivity analyses of the pharmacokinetics of PA and NAPA showed the inhibitory effects of cimetidine via rMATE1 were probably important for the renal elimination of PA and NAPA in rats. The proposed PBPK model may be useful for understanding the mechanisms of interactions between PA/NAPA and cimetidine in vivo.

## 1. Introduction

Procainamide (PA), classified as a class IA anti-arrhythmic drug, is commonly used for the long-term management of supraventricular and ventricular arrhythmias [1]. When PA was orally administered to humans, it was reported that the systemic clearance of PA was approximately 10.0 mL/min/kg [2,3,4,5,6,7], and the fraction of dose excreted via renal elimination was ranged from 29% to 65% of the systemic total clearance. Considering that the theoretical glomerular filtration rate (GFR) of PA (i.e., $f_{u}$·GFR) was estimated to be 1.5 mL/min/kg for a healthy volunteer (70 kg body weight) [8,9], a large proportion of its renal elimination appears to be due to renal secretion. Based on the cationic characteristics of PA in biological matrices (i.e., basic compound; p*K*_a_ of 9.04), the renal basolateral uptake and apical efflux of PA by organic cation transporters (OCTs) and multidrug and toxin extrusion proteins (MATEs), respectively, are considered to play vital roles in the disposition kinetics of PA [10,11,12].

According to previous reports, interactions of PA with several transporters are evident in the disposition kinetics of PA. For example, previous studies have shown PA is an inhibitor of OCT1, OCT2 and OCT3 with *K*i values of 74, 50 and 738 µM, respectively, indicating PA is a potent inhibitor of OCT2 [13,14]. PA is also identified as a substrate of MATE1 transporter (i.e., *K_m_* value of 1230 µM) [15], and a well-known substrate of OCT2 (*K_m_* value undetermined). OCT1 and OCT3 in the liver are involved in the uptake transport of PA to hepatocytes, in which PA is metabolized to *N*-acetyl procainamide (NAPA; another anti-arrhythmic agent and the major metabolite of PA), by *N*-acetyltransferase II (NAT-II) [16,17]. Taken together, previous observations imply drug-drug interactions (DDIs) of PA involve drug transporters such as OCTs and MATEs [10,11]. Furthermore, since PA and NAPA have narrow therapeutic indices [18], their DDIs may have to be carefully monitored due to the potential result of serious health risks.

Cimetidine is an inhibitor of drug transporters in the renal tubular secretory system for various drugs. For example, the AUC (i.e., area under the curve) values of metformin, ranitidine, dofetilide, and pindolol increased by up to 1.5 fold when they were co-administered with cimetidine [19,20,21,22]. Considering that the renal clearance of cimetidine, administered intravenously to humans, has been reported to be approximately 9.25 mL/min/kg [23], which is 6.4-fold higher than the theoretical GFR of cimetidine [8,9], the possibility that those drugs could share similar pathways in the renal secretory system has been expected as one of the major causes of previously reported DDIs. As widely accepted in the literature, cimetidine, a non-specific inhibitor of OCT2 and MATE1 [24], has been reported to have an apparent *K*i value ranging from 8.6 to 73 µM for OCT2, and that of 1.1 µM for MATE1 [25,26]. Although the direct mechanism responsible for the inhibition of renal OCT2 and MATE1 in the presence of cimetidine remains unclear, a number of studies have shown that cimetidine decreased the overall clearance of PA, which may be mainly caused by the decrease of the urinary excretion of PA, a victim drug of our interest [2,27,28,29,30,31]. Despite an abundance of in vitro and in vivo data on pharmacokinetic interactions between PA/NAPA and cimetidine, no mechanistic study has been undertaken on these interactions.

The primary objective of this study, therefore, was to construct a physiological model that describes the pharmacokinetic interactions between PA/NAPA and cimetidine in rats. Systemic pharmacokinetics, urinary recovery, tissue distribution at steady state and in vitro metabolism and protein binding studies for PA/NAPA were extensively performed in the presence or absence of cimetidine in the current study. The final model proposed in this study includes the pharmacokinetics of PA and NAPA in major tissue compartments for the prediction of tissue concentrations/urinary excretion profiles as well as plasma concentration profiles in the absence or presence of cimetidine in rats. A series of our simulation studies with the model incorporating semi-mechanistic kidney compartments [32,33] facilitated our sensitivity analysis to determine which process at the level of basolateral uptake by rat organic cation transporter 2 (rOCT2) (i.e., rOCT2, homolog of hOCT2) or apical efflux by rat multidrug and toxin extrusion protein 1 (rMATE1) (i.e., homolog of hMATE1) had more impact on the pharmacokinetics of PA and NAPA.

## 2. Materials and Methods

### 2.1. Materials

Cimetidine (252.34 g/mol, purity ≥ 99.0%), procainamide (PA) hydrochloride (HCl) (271.79 g/mol, purity ≥ 98.0%; 235.33 g/mol as PA), *N*-acetylprocainamide (NAPA, 277.36 g/mol, purity ≥ 99.0%), and *N*-propionylprocainamide (291.39 g/mol, purity ≥ 99.0%; used as an internal standard (IS)), were purchased from Sigma-Aldrich (St. Louis, MO, USA). Acetic acid and triethylamine were from Sigma-Aldrich. Methanol was purchased from Honeywell Burdick and Jackson (Ulsan, Korea). Water was purified in house by aquaMAX™, ultra-pure water purification system (YL Instruments, Anyang, Korea). Male Sprague-Dawley (SD) rat liver S9 fractions and potassium phosphate buffer (PBS) (pH 7.4) were obtained from BD Gentest^TM^ (Woburn, MA, USA). All other chemicals and solvents were of reagent grade or high-performance liquid chromatography (HPLC) grade and used without further purification.

### 2.2. Experimental Animals

Male SD rats (7–8 weeks old, 220–280 g) were purchased from Nara Bio Tech., Seoul, Korea. Food and water were provided ad libitum. Rats were acclimatized for 1 week to the lab environment prior to experiments and maintained under a standard 12:12 h light and dark cycle in accordance with animal protocols. All the experiments were performed as per the Guidelines for Animal Care and Use issued by Gachon University. Experimental animal protocols used in this study were reviewed and approved by the Animal Care and Use Committee of the Gachon University (#GIACUC-R2017011, approval date on 25 May 2017).

### 2.3. The Effects of Cimetidine on the Systemic Pharmacokinetics and on the Urinary Excretions of PA and NAPA

To determine whether cimetidine affects the systemic pharmacokinetics of PA and NAPA, rats were divided into two groups: A PA control group and a PA plus cimetidine co-treatment group (*n* = 4 each). Briefly, overnight fasted male rats were anesthetized with Zoletil (20 mg/kg, intramuscular injection) and then a femoral vein and artery were surgically cannulated with polyethylene tubing (PE50; Clay Adams, Parsippany, NJ, USA), for drug administration and blood sample collection, respectively. After recovery from anesthesia, rats in both groups received intravenous bolus injections. Control rats received PA HCl at a dose of 10 mg/kg in saline while rats in the co-treatment group received PA HCl (10 mg/kg) and cimetidine (100 mg/kg) (dissolved in water at pH 6, as previously described [34]). Ten minutes after the administration of blank vehicle (control group) or cimetidine (co-treatment group), PA HCl (10 mg/kg) was intravenously administered to rats. In addition, the systemic pharmacokinetics of cimetidine alone was examined to enable temporal changes in the plasma concentration of cimetidine to be considered in our pharmacokinetic models for PA and NAPA; to do this, overnight fasted male SD rats were anesthetized and catheterized as described above. After recovery from anesthesia, cimetidine at a dose of 100 mg/kg was intravenously administered (*n* = 4). Blood samples were collected at 1, 5, 15, 30, 60, 120, 180, 240, 360, and 480 min after the PA HCl administration. To compensate for the loss of body fluid due to serial blood sampling, an identical volume of saline was intravenously replenished after each blood sampling. The collected blood was immediately centrifuged at 14,000 rpm for 15 min at 4 °C to obtain plasma, which was stored at −20 °C until analysis.

To determine the effects of cimetidine on the urinary excretions of PA and NAPA, urine samples of control and co-treatment groups were collected during the intravenous administration study at intervals of 0–2, 2–4, 4–6, 6–8 and 8–24 h after the administration of PA HCl. Volumes of the urinary samples were measured, followed by 100-fold dilution of the samples (for urinary samples obtained from 0 to 8 h) or 50-fold dilution, with distilled deionized water. All samples were then stored at −80 °C until analysis.

### 2.4. The Effects of Cimetidine on the Tissue Distribution of PA and NAPA at Steady State

To evaluate the effects of cimetidine on the tissue distribution of PA and its metabolite NAPA, tissue-to-plasma concentration ratios at steady state ($K_{p,ss}$), one of the crucial terms required in PBPK calculations, was determined for various tissues. Overnight fasted male SD rats were anesthetized and catheterized as described above. Upon recovery from anesthesia, blank vehicle or cimetidine (100 mg/kg) was intravenously injected to the control or co-treatment groups (*n* = 5 each), respectively. 10 min later, the infusion of PA HCl into the femoral vein cannula was initiated, at a maintenance dose of 2.5 mg/kg/hr to control and co-treated rats using syringe pump (model no. NE-1800, New Era Pump system Inc., Farmingdale, NY, USA), immediately after the bolus injection of PA as a loading dose (1.409 and 1.413 mg/kg of procainamide, respectively, to control and co-treated rats; estimated on the basis of apparent volume of distribution at steady state (*V*_SS_)). Blood collection was carried out at 1, 5, 15, 30, 45, and 60 min after the initiation of PA administration, and the achievement of the steady state at 60 min was confirmed by no significant difference between the plasma concentrations of PA at 30, 45, and 60 min, as determined by one-way analysis of variance (ANOVA) followed by the Tukey’s post hoc test (refer to data analysis section). Blood samples were obtained from the femoral artery at 60 min PA injection, and rats were immediately sacrificed by cervical dislocation for the collection of the major tissues (i.e., brain, kidney, heart, liver, lung, and spleen). After measuring the wet weight of tissue samples, the 2-fold volume of PBS was used to homogenate the brain and spleen samples whereas the 5-fold volume of PBS was used for heart, kidney, liver, and lung, assuming the density of all tissue samples to be unity in rats. The tissue homogenates were stored at −80 °C until analysis.

### 2.5. Determination of Plasma Protein Binding of PA using Ultra-Filtration Method

After the incubation of rat plasma containing 5 µg/mL of PA at 37 °C for 12 min, 500 µL of the plasma was transferred to an Amicon Ultra centrifugal filter unit (3K-membrane, Millipore, Carrighwohil Co. Cork, Ireland). After the centrifugal filtration at 5000 rpm for 12 min, the concentrations of PA in filtrates were determined by UHPLC system (see below). Then, the free fraction of PA in the plasma ($f_{up}$) was estimated by dividing the concentration of PA in the filtrate by the total concentration.

### 2.6. The Effect of Cimetidine on in vitro Metabolic Conversion of PA to NAPA in Rat Liver S9 Fractions

To evaluate the metabolic activity by NAT-II enzymes, the metabolic stability of PA with or without cimetidine was evaluated in vitro using rat liver S9 fractions [35]. Reaction mixtures consisted of rat liver S9 fractions (2 mg protein/mL), 100 mM potassium phosphate buffer, and an NADPH regenerating system, containing PA (final concentrations in reaction mixtures: 50, 500, and 5000 µM) with or without cimetidine (final concentrations of 0, 1, or 5 mM, at the three PA concentrations, respectively). The mixture was preincubated at 37 °C for 5 min in a Multi-Therm Shaker (Benchmark Scientific, South Plainfield, NJ, USA) set at 200 oscillations per min. After preincubation, the reaction was started by adding 10 µL of 20 mM acetyl-CoA and vortex mixing. Upon collecting samples at 0 min and 30 min after initiation by pipetting out 50 µL of aliquot into an Eppendorf tube, the metabolic reaction was terminated by adding 100 µL of ice-cold methanol containing IS (200 ng/mL). To determine the *IC*_50_ value for the inhibitory effect of cimetidine on the conversion of PA to NAPA, reaction mixtures of rat liver S9 fractions containing PA (final concentration of 50 µM) with or without cimetidine (at final concentrations of 0, 0.5, 1, 5, 10, 50, 100, 500, 1000, or 5000 µM) were separately prepared in triplicate. After the preincubation of the mixture (37 °C for 5 min in Benchmark Multi-Therm Shaking Vortexer set at 200 oscillations per min), the reaction was initiated by adding 10 µL of 20 mM acetyl-CoA and vortex mixing. Samples (50 µL) were collected at predetermined times (0, 15, or 30 min after initiation) and ice-cold methanol (100 µL) containing IS (200 ng/mL) was added to terminate the reaction. All samples were vortex mixed and centrifuged at 12,000 g for 10 min at 4 °C. Finally, 70 µL aliquots of supernatants were transferred into vials and 2 µL was injected into the UHPLC system to determine the concentration of the formed metabolite, NAPA.

### 2.7. Bioanalytical Condition

The concentrations of PA and NAPA in various matrices (e.g., plasma, urinary samples, and samples from in vitro studies) were determined as previously described [36]. Briefly, analyses were performed with an Agilent Technologies 1290 Infinity II UHPLC system (Agilent Technologies, Palo Alto, CA, USA) equipped with a multisampler (G7167B), a flexible pump (G7104A), a multicolumn thermostat (MCT) (G7116B), and a DAD detector (G7117A). A Synergi polar–RP column 80A (150 × 2.0 mm, 4 µm; Phenomenex, Torrnce, CA, USA) column was used to separate PA, NAPA and IS. The mobile phase used was composed of 1% acetic acid (pH 5.5) and methanol (76:24, *v*/*v*), and the elution was performed in an isocratic mode at a flow rate 0.2 mL/min. The injection volume was 2 µL, and the detection wavelength was 280 nm. The column and autosampler tray were maintained at 25 and 4 °C, respectively.

To determine cimetidine concentrations, we used a previously described LC-MS/MS assay [37] with minor modification. Briefly, 50 µL of plasma was vortexed with methanol (200 µL), followed by centrifugations at 16,100× g for 5 min at 4 °C. Supernatant (10 µL) was directly injected onto the LC-MS/MS system (Applied Biosystems 3200 Qtrap MS/MS system (Applied Biosystems, Foster City, CA, USA) equipped with Alliance Waters e2695 LC system (Waters Corporation, Milford, MA, USA)) using the following operating condition: ion spray voltage, 5500 V; curtain gas, 10 (arbitrary units); GS1 and GS2, 50 and 40 psi, respectively; probe temperature, 600 °C; declustering potential, 21 V; entrance potential, 6 V; collision energy, 31 eV; and collision cell exit potential, 4 V. Electrospray ionization was used in positive ion mode, and nitrogen was used as the nebulizer and the collision gas. The isocratic mobile phase consisted of 0.1% formic acid in acetonitrile and 0.1% formic acid in water (80:20, *v*/*v*), and was delivered at a flow rate of 1 mL/min at 25 °C to a reverse phase HPLC column (Agilent Poroshell 120, EC-C18 2.7 µm, 4.6 × 50 mm). Cimetidine was analyzed in MRM mode at *m*/*z* 253.1 to *m*/*z* 95.2. Throughout the assay, the temperature of the autosampler compartment was maintained at 4 °C.

### 2.8. Data Analysis

#### 2.8.1. Noncompartmental Pharmacokinetic Analysis

In this study, conventional noncompartmental analyses were conducted using Winnonlin Professional 5.0.1 software (Pharsight Corporation, Mountain View, CA, USA) to calculate pharmacokinetic parameters [38], including area under the plasma concentration-time curve (*AUC*) from time zero to infinity (*AUC*_inf_), systemic clearance (*CL*), terminal phase half-life, mean residence time (*MRT*), and volume of distribution at steady state (*V*_SS_). In addition, parameters including the maximal concentration (*C*_max_) and the time to the *C*_max_ of NAPA (*T*_max_) were also estimated.

When necessary, renal clearances (*CL*_R_) of PA and NAPA were calculated, assuming their urinary recoveries of PA and NAPA were completed at 24 h after the administration of PA HCl, as follows (Equation (1)):

(1) $$CL_{R}=\frac{X_{e,0-24h}}{AUC_{\inf,\mathrm{plasma}}}$$

where $X_{e,0-24h}$ is the cumulative amount of PA excreted in the urine, from time zero up to 24 h after PA HCl administration, and $AUC_{\inf,\mathrm{plasma}}$ is the area under the plasma concentration-time curve from time zero to infinity. Non-renal clearance (*CL*_NR_) was then determined by subtracting *CL*_R_ from *CL*. Based on the assumption that PA and NAPA were eliminated only via kidney and liver, *CL*_NR_ was considered identical to the hepatic clearance (*CL*_H_).

The tissue-to-plasma concentration ratios at steady state ($K_{p,ss}$) of PA and NAPA, were calculated by the ratio of the drug concentrations in tissues to those in plasma, based on the assumption that drug concentrations in the six major tissues (i.e., brain, kidney, heart, liver, lung, and, spleen) had achieved a steady state at the tissue sampling time. For eliminating organs (i.e., kidney and liver), $K_{p,ss}$ values were corrected to the equilibrium tissue-to-plasma partition coefficient ($K_{p}$), the parameter that remains unchanged regardless of drug elimination rate [39], because of the considerable extraction ratios of PA and NAPA, using the following relationship (Equation (2)):

(2) $$K_{p,ss}=\left(1-ER\right)\cdot K_{p}$$

where ER is the extraction ratio, which was calculated by dividing organ (blood) clearance (*CL*_org_) by the blood perfusion rate ($Q_{T}$) to the organ.

AUC values with respect to the plasma concentrations of PA (*AUC*_PA_) and NAPA (*AUC*_NAPA_) after the intravenous injection of PA were determined by noncompartmental analysis, and used to calculate the formation clearance ($CL_{m}$) from PA to NAPA using the following relationship (Equation (3)) [40]:

(3) $$CL_{m}=\frac{AUC_{\mathrm{NAPA}}}{AUC_{\mathrm{PA}}}CL_{\left(m\right)}$$

where $CL_{\left(m\right)}$ is the disposition clearance of NAPA. The fraction of NAPA formation (*F*_NAPA_) during the hepatic elimination of PA was then calculated.

#### 2.8.2. In Vitro Kinetic Analysis

The amounts of NAPA formed from PA in reaction mixtures were determined using the rat liver S9 fractions. When it was necessary to analyze in vitro kinetic parameters for the concentration-dependent inhibition of the metabolic conversion of PA to NAPA by cimetidine, the following equation (Equation (4)) was fitted to in vitro data to estimate the half maximal inhibitory concentration (*IC*_50_):

(4) $$v=\frac{v_{max}}{1+\frac{\left[I\right]}{IC50}}$$

where v is the metabolic conversion rate of from PA to NAPA, $v_{max}$ is the maximal metabolic rate, [I] is inhibitor concentration in the reaction mixture. The percent activity for the metabolic rate of PA in the presence of cimetidine was calculated considering the rate in the absence of cimetidine as 100% of the activity.

### 2.9. Physiologically-Based Pharmacokinetic Model for PA, NAPA, and Cimetidine

In this study, a mechanistic approach with physiologically-based pharmacokinetic (PBPK) modeling was used for the prediction of drug-drug interactions between PA/NAPA and cimetidine. When it was necessary, kinetic parameters for the substrate (PA) and its metabolite (NAPA) were calculated using a series of model refinement processes, based on the experimental results including plasma concentrations and urinary excretion profiles of PA and NAPA after a single intravenous dose of PA HCl (10 mg/kg). In addition, pharmacokinetic parameters for the inhibitor (cimetidine) were obtained based on plasma concentrations after 100 mg/kg of cimetidine was intravenously administrated. To determine whether the pharmacokinetics of PA and NAPA was affected by the co-administration of cimetidine, temporal changes in cimetidine concentrations in involved compartments (e.g., free concentration in plasma and renal proximal tubule cells) were used for the prediction of PA/NAPA pharmacokinetics, using in vitro *K*i values of cimetidine on the basolateral uptake via rOCT2 and the apical efflux of a representative substrate (see below).

**Figure 1:**
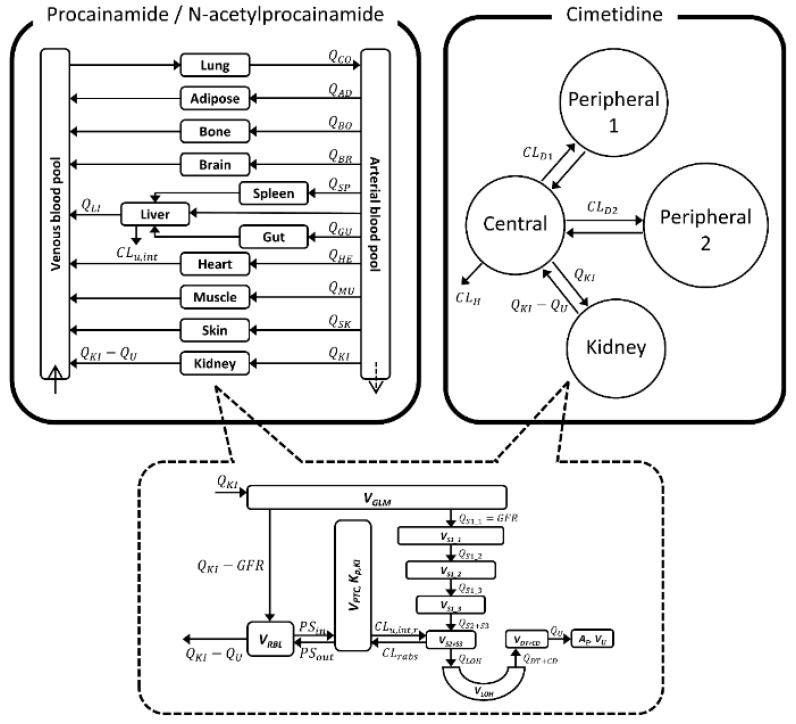
Structure of the whole-body physiologically-based pharmacokinetic (PBPK) model used to describe the pharmacokinetics of procainamide (PA) and *N*-acetylprocainamide (NAPA) (left upper), and of the minimal PBPK model used for cimetidine (right upper), were incorporated into the final PBPK model with a semi-mechanistic kidney model (lower).

The structure of our PBPK model is shown in Figure 1. Whole-body PBPK models with 11 major tissues were used for the kinetics of PA and NAPA, whereas a minimal PBPK model was used for cimetidine. For kidney, a major organ where DDIs may occur, a previously described semi-mechanistic model was incorporated in the present study [32,33]. Physiological and anatomical variables, required in PBPK calculations, were obtained from the literature [41] (i.e., essentially the default values found in Simcyp software [42], version 15, release 1; Simcyp Limited, Sheffield, UK), as summarized in Table 1. When necessary for the numerical simulations of the models, computations were carried out with Berkeley Madonna software (version 8.3.18; University of California, Berkeley, CA, USA). In this study, the fourth order of the Runge-Kutta method was used for numerical integration.

#### 2.9.1. PA and NAPA

The information used in the development of a PBPK model for PA and NAPA in rats is summarized in Table 2. For PA and NAPA, the PBPK model consisted of eleven major tissues (i.e., adipose, bone, brain, gut, heart, kidney, liver, lung, muscle, skin, and spleen), which were assumed to be connected to the circulatory system (i.e., arterial and venous blood compartments). In the PBPK calculation, the rate of PA and NAPA distribution to tissues was assumed to be perfusion-rate limited and the corresponding standard mass balance differential equations were used [44,48] (see Appendix A).

$K_{p}$ values of PA for the six major tissues (i.e., brain, kidney, heart, liver, lung, and, spleen) were experimentally determined in this study, and those values for other tissues (i.e., adipose, bone, gut, muscle, and skin) were predicted as previously described [49,50].

Since the systemic clearance (*CL*) and renal clearance (*CL*_R_) of PA with respect to plasma concentration was determined after the intravenous dose of PA HCl in this study, the hepatic clearance was assumed to be identical to the non-renal clearance (*CL*_NR_), which was estimated by subtracting *CL*_R_ (13.4 mL/min/kg) from *CL* (73.3 mL/min/kg), based on the assumption that PA was eliminated only in kidney and liver. Using *CL*_R_ (15.2 mL/min/kg) as determined by the noncompartmental analysis of this study, *CL*_NR_ (7.20 mL/min/kg) was estimated since the systemic clearance of NAPA (20.7 to 22.4 mL/min/kg) was determined when NAPA was intravenously administered in previous studies [51,52]. According to the literature [28], blood-to-plasma concentration ratio (R) of PA and NAPA in rats was considered to be unity, assuming that there is little species difference in the parameter (i.e., human R of 0.98). The free fraction of PA in rat plasma was determined using the ultrafiltration method in the current study ($f_{up}=0.870$). Using the conventional well-stirred liver model [53], the unbound intrinsic clearance ($CL_{u,int}$) for the hepatic elimination was then calculated as follows:

(5) $$CL_{u,int}=\frac{Q_{H}R\cdot CL_{H}}{f_{up}\left(Q_{H}R-CL_{H}\right)}$$

where $Q_{H}$ is the hepatic blood flow.

In the present study, the semi-mechanistic kidney model [32,33] was incorporated in the PBPK model, based on the considerations of physiologically-relevant fluid reabsorptions and carrier-mediated transports of PA and NAPA (see Appendix A). Briefly, rat kidney was composed of a series of nephron segments (i.e., glomeruli, proximal tubules, loops of Henle, distal tubules, and collecting ducts), in which segmental fluid flow rates and volumes were applied as functions of GFR (Figure 1, Table 1). For the renal excretion kinetics of PA, GFR with regard to free molecules (i.e., $f_{up}$·GFR/R, 4.56 mL/min/kg) was estimated using a physiological GFR value in rat kidney (i.e., 5.24 mL/min/kg) [9], indicating the involvement of the renal secretion process of PA into urine (i.e., for PA, *CL*_R_ of 13.4 mL/min/kg). The renal secretion process of NAPA was also considered since $f_{up}\cdot \mathrm{GFR}/R$ was calculated to be 3.61 mL/min/kg while *CL*_R_ was determined to be 15.2 mL/min/kg. In addition, partial reabsorption of PA in proximal tubules was considered (viz, fraction of renal reabsorption, *F*r = 0.490), since our preliminary simulation overestimated the proportion of the cumulative amount of PA excreted into urine (from time 0 to 24 h) when the secretion process in our primary model was truncated (data not shown). In addition, it was assumed that *F*r of NAPA was the same as that of PA, in the current study.

In this study, passive diffusional clearance of PA, essentially a permeability-surface area product of PA from kidney tissue (i.e., proximal tubule cells) to renal blood compartment ($PS_{out}$), was obtained by multiplying PAMPA (i.e., parallel artificial membrane permeability assay) permeability (0.310 × 10^−6^ cm/s) [54] by the effective surface area values ($S_{eff}$) for proximal tubule cells, as described previously [44]. On the basis of the similar physicochemical properties of PA and NAPA (Table 2), we assumed PAMPA permeability of NAPA was assumed the same as that of PA. The free fraction of PA and NAPA in the kidney ($f_{u,kidney}$) was predicted by Rodgers and Rowland’s method [49]. By assuming that the kidney-to-plasma concentration ratio for PA and NAPA when the distribution kinetics is mainly governed by passive diffusion (i.e., $K_{p,KI,pass}$) could be reasonably represented by the ratio of $f_{up}$ to the predicted $f_{u,kidney}$, $K_{p,uu}$ (i.e., equilibrium unbound tissue-to-unbound plasma concentration ratio; $K_{p,uu}=PS_{in}/PS_{out}$) was estimated by dividing the $K_{p}$ values of PA and NAPA in kidney by $K_{p,KI,pass}$. $PS_{in}$, passive and active distributional clearance from renal blood compartment into kidney tissue, was then estimated using predicted $K_{p,uu}$ and $PS_{out}$ values.

Using the plasma concentration and urinary excretion profiles of PA, the renal intrinsic clearance ($CL_{u,int,r}$) (see Appendix A), which is related to renal efflux of PA into the urine, was optimized by Winnonlin Software. After the determination of the $CL_{u,int,r}$ value for PA, the $CL_{u,int,r}$ value for NAPA was also fitted to the observed profile of NAPA after the intravenous administration of 10 mg/kg PA HCl.

#### 2.9.2. Cimetidine

In the case of cimetidine, the minimal PBPK model [55] (Figure 1; see Appendix A), incorporating the semi-mechanistic kidney was applied (as applied in the PA and NAPA model, see below). The information used for the PBPK modeling of cimetidine is summarized in Table 3. Blood binding characteristics of cimetidine, including the free fraction in rat plasma ($f_{up}$ = 0.836) and R-value (R = 1), were obtained from the literature [56,57], on the basis of the assumption of little species difference in R-value (human R = 0.97). According to the goodness-of-fit criteria including visual inspections of fitted curves, the Akaike information criterion (AIC) and the coefficient of variation (CV), the central compartment with two peripheral compartments was considered to be appropriate for the cimetidine pharmacokinetics, and consequent parameters required for the PBPK calculation (e.g., distributional clearance, and volume of distribution for tissue compartments) were obtained using Winnonlin software.

As described for PA and NAPA, previously reported PAMPA permeability of cimetidine (3.02 × 10^−6^ cm/s) [61] was used for the prediction of $PS_{out}$. In this study, the kidney-to-plasma concentration ratio of cimetidine at 90 min after a bolus injection of 8 mg/kg cimetidine (9.25) [60] was regarded as the $K_{p,ss}$ of kidney tissue. Since the fraction of the dose excreted unchanged for cimetidine (*f*e = 0.45) was determined with the same dose (100 mg/kg) in the literature [64], *CL*_R_ for cimetidine could be reasonably calculated by multiplying the systemic clearance (20.3 mL/min/kg) by *f*e for cimetidine. The extraction ratio of cimetidine in kidney tissue was then determined by dividing the *CL*_R_ of cimetidine by the blood perfusion rate to kidney tissue, to use the parameter for the correction of $K_{p,ss}$ into $K_{p}$ (Equation (2)). Similarly, for PA and NAPA, $K_{p,KI,pass}$ was predicted by Rodgers and Rowland’s method [50], which was then used for the calculation of $K_{p,uu}$ for cimetidine in the kidney. $PS_{in}$ was estimated using $K_{p,uu}$ and $PS_{out}$ values predicted for cimetidine. Assuming no reabsorption process in the renal excretion of cimetidine, $CL_{sec}$ (4.76 mL/min/kg) was obtained by subtraction of $f_{up}$·GFR from *CL*_R_ [11], to calculate $CL_{u,int,r}$ required for renal secretion process in the conventional well-stirred model, as follows (Equation (6)):

(6) $$CL_{u,int,r}=\frac{Q_{K}R\cdot CL_{sec}}{f_{up}\left(Q_{K}R-CL_{sec}\right)}$$

After the model refinement process for each drug, the information on the inhibition kinetics of cimetidine on the transporter activity (e.g., rOCT2 and rMATE1) was collected from the literature. *K*i values for cimetidine on [^14^C]-tetraethylammonium (i.e., TEA, a representative substrate) accumulation via rOCT2 were used to predict its interactions with PA and NAPA (*K*i of 9.4 μM) [62]. Since it was reported that cimetidine transport mediated by rMATE1 was saturable with a *K_m_* value of 3.01 µM [63], *K*i value of cimetidine in this study was considered to be equal to *K_m_* based on the assumption of the competitive inhibition of cimetidine on the PA and NAPA transport via rMATE1.

### 2.10. Statistics

When it was necessary to evaluate the predictive performance of the PBPK model for DDIs between PA/NAPA and cimetidine, the absolute average fold error (AAFE) was calculated by comparing the model predicted concentrations/amounts with the observed values as follows (Equation (7)):

(7) $$AAFE=10^{\frac{1}{n}\sum \left|log\frac{C_{pred}}{C_{obs}}\right|}$$

where $C_{pred}$ and $C_{obs}$ are the predicted and observed concentrations, and n is the number of observed points.

To compare the means among groups, the two-tailed unpaired Student’s *t*-test or one-way ANOVA followed by the Tukey’s post hoc test, was used. In this study, data are presented as means ± standard deviation and statistical significance was accepted with *p*-values less than 0.05.

## 3. Results

### 3.1. The Effects of Cimetidine on the Systemic/Tissue Pharmacokinetics and the Urinary Excretions of PA and NAPA

**Figure 2:**
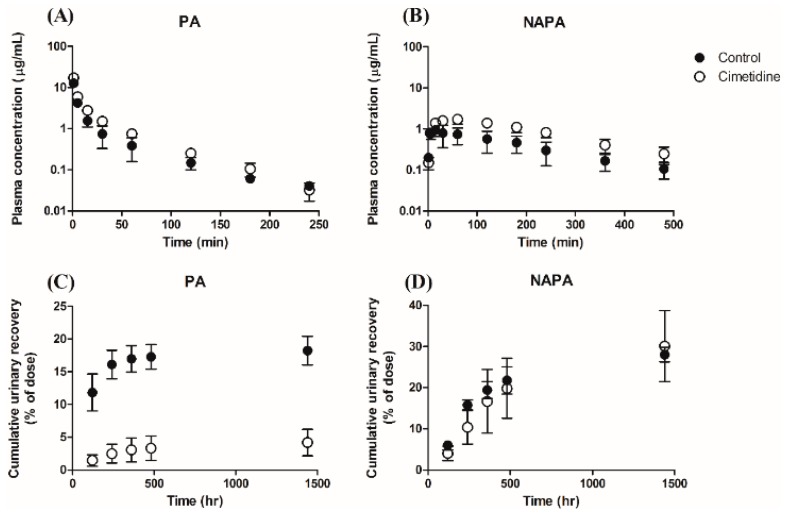
Temporal profiles of plasma concentrations of (**A**) PA and (**B**) NAPA, and the cumulative urinary excretions of (**C**) PA and (**D**) NAPA, after 10 mg/kg dose of PA HCl in rats, in the absence or presence of cimetidine (10 min before PA administration). Closed and open circles represent results observed without (control) or with cimetidine. Symbols represent the mean ± S.D (*n* = 4, for each group).

For the systemic pharmacokinetics, the mean plasma concentration profiles are presented in Figure 2A (for PA) and Figure 2B (for NAPA). When an intravenous bolus dose of 10 mg/kg PA HCl was administered to rats in the absence or presence of 100 mg/kg cimetidine (10 min prior to PA dose), cimetidine co-administration increased the plasma concentration of PA. The pharmacokinetic parameters determined by non-compartmental analysis are summarized in Table 4. The observed CL of PA without or with cimetidine was 73.3 ± 11.3 mL/min/kg or 45.1 ± 3.64 mL/min/kg (*p* < 0.001), respectively, and the *CL*_R_ values of PA and NAPA were 13.4 ± 2.07 mL/min/kg and 15.2 ± 4.86 mL/min/kg without cimetidine, respectively. However, the *CL*_R_ values of PA and NAPA were dramatically decreased in the presence of cimetidine (1.89 ± 0.152 mL/min/kg (*p* < 0.001) and 6.51 ± 2.24 mL/min/kg (*p* < 0.05)), respectively (Table 4). Collectively, these results indicate that the renal elimination processes of PA and NAPA lead to DDIs when PA and cimetidine are co-administered to rats. The clearance terms obtained in this study were comparable to those previously reported [51,52]. Secondary pharmacokinetic parameters (e.g., *AUC*_inf_, terminal phase half-life, and mean residence time) of PA and NAPA were also evaluated (Table 4).

The urinary excretion profiles of PA and NAPA in the absence or presence of cimetidine after a single dose of 10 mg/kg PA HCl are shown in Figure 2C (for PA) and 2D (for NAPA), respectively. In the presence of cimetidine, the cumulative urinary excretion of PA recovered up to 24 h significantly decreased from 18.2 ± 2.18% to 4.17 ± 2.01% of PA dose administered. However, for the urinary excretion of NAPA, the cumulative urinary recovery up to 24 h after PA injection in the absence or presence of cimetidine was 28.0 ± 1.51% and 30.1 ± 8.63%, respectively, which were not significantly different from each other.

The *V*_SS_ of PA in the absence or presence of cimetidine was 2430 ± 539 mL/kg or 1520 ± 133 mL/kg, respectively (Table 4), indicating that cimetidine decreased the tissue distribution of PA in rats. Consistent with these results, $K_{p,ss}$ of PA in tissues including heart, liver, and lung tissues at steady state was also decreased by cimetidine co-administration (Table 5). In addition, $K_{p,ss}$ of NAPA in 6 major tissues obtained was also decreased by cimetidine co-administration (Table 5).

### 3.2. The Effect of Cimetidine on In Vitro Metabolic Conversion of PA to NAPA in Rat Liver S9 Fractions

**Figure 3:**
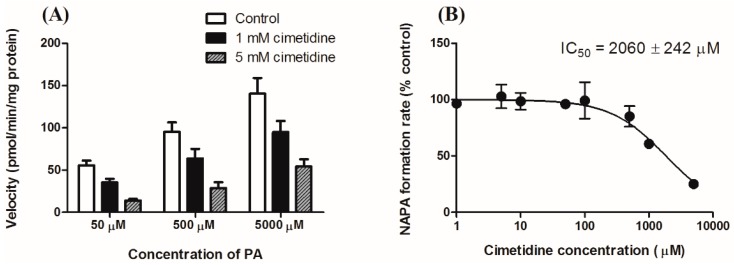
Effects of cimetidine on the metabolic conversion of PA to NAPA using in vitro rat liver S9 fraction. (**A**) Dependence of the metabolic conversion rate on the PA concentration in rat liver S9 fractions (50, 500, and 5000 μM) (*n* = 5 for each column) (**B**) Inhibitory effects of cimetidine on the conversion rate of PA to NAPA in rat liver S9 fractions (three independent experiments with triplicate measurement) (*n* = 3 for each point). Symbols represent the mean ± S.D.

In this study, inhibitory effects of cimetidine on the formation kinetics from PA to NAPA was evaluated using in vitro S9 fractions obtained from rat liver. The formation rate of NAPA from PA was determined by incubating rat liver S9 fractions with PA at concentrations ranging from 50 to 5000 μM with or without cimetidine (1 or 5 mM) as shown in Figure 3A. The *IC*_50_ value for the inhibitory effect of cimetidine on the formation kinetics of NAPA from PA was observed to be 2060 ± 242 μM (Figure 3B).

### 3.3. Development of a PBPK Model for the Pharmacokinetics of PA and NAPA after a Single Intravenous Dose of PA in the Absence or Presence of Cimetidine

**Figure 4:**
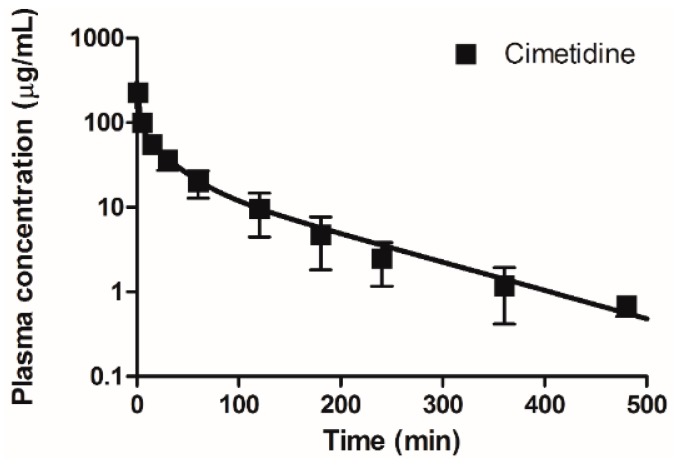
Observed and simulated (i.e., model fitted) plasma concentration-time profiles for cimetidine after intravenous bolus administration of 100 mg/kg of cimetidine. Closed squares and the solid line represent the observed and simulated cimetidine plasma concentrations, respectively (*n* = 4 for each point).

**Figure 5:**
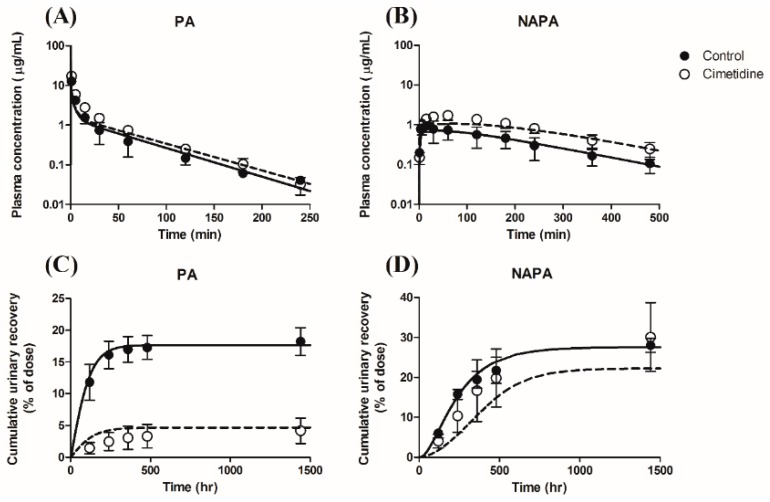
Observed and simulated plasma concentration-time profiles (**A**,**B**) and urinary excretion profiles (Cumulative urinary recovery (% dose)) (**C**,**D**) for PA (**A**,**C**) and NAPA (**B**,**D**) after intravenous bolus administration of 10 mg/kg of PA HCl in the absence or presence of cimetidine. (Observed results from Figure 2). Closed circles and solid lines represent the observed and simulated (model fitted) results without cimetidine, whereas open circles and dashed lines represent the observed and simulated (predicted) results with cimetidine, respectively. Symbols represent the mean ± S.D.

In this study, a PBPK model (Figure 1) was developed to predict the plasma concentrations and urinary excretion profiles of PA and NAPA in the absence or presence of cimetidine, using the pharmacokinetic parameters obtained from in vitro/in vivo/in silico data (Table 2 and Table 3). Model parameters were determined using a model refinement process for each drug, resulting in reasonable model predictions for PA and NAPA (after a single dose of PA), and for a single dose of cimetidine, respectively (Figure 4 and Figure 5).

Assuming that the refined PBPK model is predictive for each drug, it may be used to predict in vivo DDIs between PA/NAPA and cimetidine, by using pharmacokinetic parameters related to the potential DDIs (Table 2 and Table 3). The observed and simulated plasma concentrations and urinary excretion profiles when PA was co-administered with cimetidine to rats were presented in Figure 5. The simulated profiles for DDIs between PA/NAPA and cimetidine resulted in AAFE values (without and with cimetidine, respectively) within a factor of two for the plasma concentrations of PA (1.36 and 1.43) and NAPA (1.23 and 1.48), respectively, and for the urinary excretion profiles of PA (1.04 and 1.53) and NAPA (1.12 and 1.65), respectively, indicating that the devised PBPK model showed a reasonable performance for the prediction of DDIs between these drugs.

**Figure 6:**
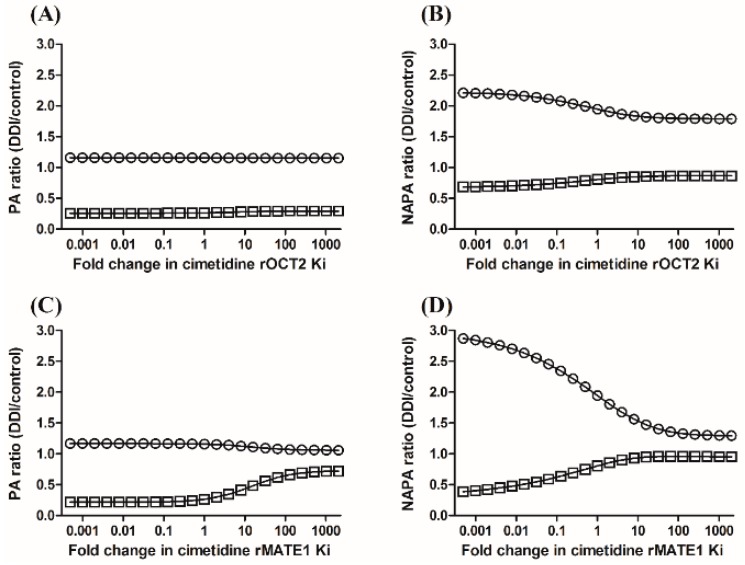
Sensitivities of the PA ratios (**A**,**C**) and NAPA ratios (**B**,**D**) of plasma AUC (circles) and urinary recovery (squares) to cimetidine *K*i values for rOCT2 (**A**,**B**) and rMATE1 (**C**,**D**), based on a PBPK model incorporating a semi-mechanistic kidney compartment.

When PA was co-administered with cimetidine, the AUC ratios (i.e., *AUC*_DDI_/*AUC*_control_) of PA and NAPA in the plasma were 1.60 and 2.42, respectively, whereas the ratios of *CL*_R_ (i.e., *CL*_R,DDI_/*CL*_R,control_) of PA and NAPA were 0.141 and 0.428, respectively (Table 4). Using the PBPK model proposed in this study, sensitivity analysis indicated that the changes of the plasma AUC ratio caused by the inhibition of either rOCT2 or rMATE1 were neglectable, whereas the urinary excretion of PA was more sensitive to the changes of *K*i values of cimetidine for rMATE1 than that for rOCT2 (Figure 6A,C). The plasma AUC ratio and urinary excretion of NAPA were more sensitive to the changes of *K*i values of cimetidine for rMATE1 than that for rOCT2 (Figure 6B,D).

## 4. Discussion

According to the literature [2,27,28,29,30,31], pharmacokinetic interactions between PA/NAPA and cimetidine reduced the systemic clearance (*CL*) of PA and NAPA when cimetidine was co-administered with PA in humans. It is noteworthy that the renal clearance (*CL*_R_) of PA was mainly decreased by cimetidine co-administration by 36.0% to 43.4% of the values obtained without cimetidine [2,28,29]. Despite an abundance of in vitro and in vivo data regarding pharmacokinetic interactions between PA/NAPA and cimetidine, a mechanistic approach to elucidate the drug interaction based on the considerations of rOCT2 and rMATE1 was not found so far. To the best of our knowledge, the current study is the first to investigate the pharmacokinetics of PA and NAPA simultaneously in the absence or presence of cimetidine to rats, using a PBPK modeling approach.

As summarized in Table 4, the *CL* (73.3 ± 11.3 mL/min/kg) and the *CL*_R_ (13.4 ± 2.07 mL/min/kg) of PA obtained in this study were comparable with the previously reported values (i.e., *CL* of 80.7 mL/min/kg and *CL*_R_ of 32.0 mL/min/kg), whereas *CL*_R_ values for NAPA (15.2 ± 4.86 mL/min/kg) determined in this study were well compared with the literature value (14.7 mL/min/kg) [52]. In addition, the terminal phase half-lives of PA (50.4 ± 8.38 min) and NAPA (153 ± 15.6 min) were comparable with the reported value of 39.6 min and 133 min, respectively. The apparent volume of distribution of PA (2.43 ± 0.539 L/kg) was also considered comparable with the results from the literature (e.g., 4.60 to 4.92 L/kg) [51,52]. Collectively, the pharmacokinetic properties of PA and NAPA determined in the present study were consistent with previously reported values.

One of the primary observations of the current study was that co-administration of cimetidine, a rOCT2 and rMATE1 inhibitor, significantly increased the AUC and decreased the *CL* and *CL*_R_ values of PA, and its tissue distribution. In addition, the *CL*_R_ of NAPA was also significantly decreased in the presence of cimetidine, resulting in increased AUC of formed NAPA. Based on the possibility that these drugs could share similar pathways in the renal secretory system, we hypothesized that rOCT2 and rMATE1 might play crucial roles in the DDIs observed in the current study. Since rOCT2 and rMATE1 are located on the basolateral and apical membranes of renal proximal tubule cells, respectively [11], a PBPK modeling approach was considered suitable to improve understanding of the observed DDIs between the two victims (PA and NAPA) and a perpetrator (cimetidine) at each level of the elimination process. Therefore, the active transport via these transporters was incorporated into our kidney model (Figure 1): As mathematically described in Equations (A21) and (A22) (see Appendix A), the inhibitory effect of cimetidine on the active transport via rOCT2 was reflected in the disposition kinetics of both PA and NAPA (e.g., $K_{p,KI}$), while the apical efflux of the victim drugs, expressed as the renal intrinsic clearance ($CL_{u,int,r}$) was considered to be inhibited by free cimetidine in the renal proximal tubule cell compartment (Equation (A23)). Despite assumptions regarding experimental values obtained from in vitro studies for the inhibition of these active transport processes (Table 3), the adequate performance of the final PBPK model for the prediction of DDIs (Figure 5) suggested that the observed DDIs between PA/NAPA and cimetidine in rats are mainly due to the inhibition of the transport process of PA and NAPA via rOCT2 and rMATE1. In clinical situations, PA and cimetidine are generally administered as oral formulations. The increased systemic exposure of PA by the cimetidine co-administration was previously reported likely due to the reduced oral clearance [27]. Since this present study originally focused on the elucidation of the mechanism of DDIs between PA/NAPA and cimetidine in the renal elimination process, further studies for the mechanistic approach to the elucidation of DDI mechanism in the oral absorption process may be warranted.

During our PBPK model refinement process, the perfusion-limited model was adopted for the distribution kinetics of PA and NAPA in rats. Recently, the minimum permeability coefficient from the systemic circulation to tissues was estimated for the perfusion-limited distribution of drugs in rats, assuming that in vitro PAMPA permeability could reasonably predict in vivo tissue permeability [44]. Using a reported PAMPA permeability ($P_{app,PAMPA}$) of PA through the artificial membrane consisting of 2% phosphatidylcholine [54], and the effective surface area of the proximal tubule cell compartment [44], the in vivo apparent PAMPA permeability via passive transport (i.e., $f_{up}P_{app,PAMPA}/R$) was estimated to be 0.270 × 10^−6^ cm/s, which is slightly smaller than the proposed threshold (i.e., 1 × 10^−6^ cm/s) for perfusion-limited distribution. Since our preliminary simulations showed that the predictions for the systemic pharmacokinetics of PA were scarcely affected by the permeability coefficient (data not shown), we considered the perfusion-limited model applicable for the distribution kinetics of PA. Despite a lack of the information on the relevant PAMPA permeability of NAPA (i.e., a structural analogue of PA with similar lipophilicity) in the literature, our sensitivity analysis also suggested the use of the perfusion-limited model for the distribution kinetics of NAPA since the permeability coefficient of NAPA has little impact on the plasma concentration of NAPA, providing an indistinguishable profile with in vivo data (data not shown).

Whereas the liver compartment for PA and NAPA was described by a typical well-stirred model, a modified semi-mechanistic model was used for kidney tissue [32,33]. The perfusion-limited model may also be useful for the distribution of PA and NAPA to the kidney due to their sufficient permeability characteristics, but this semi-mechanistic model that is much closer to the physiological situation was considered to be useful for the description of observed sigmoidal increases in the cumulative urinary recovery of drugs (Figure 5C,D). Assuming that the prediction method for tissue permeability via passive transport [44] is appropriate for PA and NAPA, we predicted efflux tissue permeability from the eliminating organs ($PS_{out}$) (Table 2), in which the effective surface area normalized by kidney weight was multiplied by the volume of proximal tubule cell compartment. Since the equilibrium tissue-to-plasma concentration ratio ($K_{p}$) [39] can be determined by the correction of $K_{p,ss}$ of eliminating organ with extraction ratio ($ER=CL_{org}/Q_{T}$), uptake tissue permeability ($PS_{in}$, a summation of passive and active drug uptake), could also be estimated using $K_{p,uu}$ (i.e., $PS_{in}/PS_{out}$). In this study, $K_{p,uu}$ was calculated by dividing $K_{p}$ by $K_{p,pass}$ predicted using Rodgers and Rowland’s method [49], and assuming that the major binding characteristics can be reasonably predicted by the method. Due to the partial reabsorption process considered for PA and NAPA in this study, $CL_{u,int,r}$ was then optimized to the experimentally observed data. The resulting $CL_{u,int,r}$ values of PA and NAPA were 4.67 and 9.16 mL/min, respectively, compared with the predicted $PS_{out}$ (7.61 mL/min), suggesting the possible asymmetry of passive diffusional clearance in the process of basolateral and apical drug transport. In an alternative approach to predict $PS_{out}$ of PA, we used in vitro uptake rate of ^14^C-labeled PA into HEK293 cells transfected with empty vector (10.4 μL/mg protein/0.5 min) [65]. Using this approach, $PS_{out}$ was predicted to be 4.54 mL/min based on the protein level of rat kidney tissue reported in the literature (212 mg protein/g kidney) [66], indicating that both methods provided quite comparable predictions for tissue permeability via passive transport in the rat kidney. In addition, a series of calculations were also conducted for the pharmacokinetic parameters used for NAPA for our PBPK simulations (Table 2).

Through the typical moment analysis for plasma concentration profiles of PA and NAPA, the formation clearance from PA to NAPA with regard to plasma concentration of PA could be calculated in vivo (8.01 mL/min), calculated from Equation (3). The estimation of F_NAPA_ in the hepatic elimination process of PA facilitated our calculation of the unbound intrinsic clearance for NAPA formation process (11.8 mL/min), using $f_{u,liver}$ predicted by Rodgers and Rowland’s method. In this study, the rate of metabolic formation from PA to NAPA was determined in the rat liver S9 fractions (50.4 ± 4.63 pmol/min/mg protein). Considering the protein level of the S9 fraction and the wet weight of the rat liver (165 mg protein/g liver) [67], the unbound intrinsic clearance for the formation process in vivo could be estimated (1.43 mL/min), resulting in the additional scaling factor of 8.31. One of the possible reasons for this discrepancy may be attributed to a lack of consideration of protein binding of PA in the reaction mixture of S9 fractions. Though the formation clearance of NAPA from PA determined in vivo was adopted for our PBPK calculations, further studies on in vivo-in vitro extrapolation (IVIVE) of the formation kinetics of NAPA from PA may be warranted.

The affinity of PA for rat renal transporters has been reported, including rOCT2 with *K*i values of 167 to 748 μM [68,69]. In the present study, the free plasma concentration of PA in the plasma at a dose of 10 mg/kg PA HCl was below 47.0 μM (e.g., 11.0 μg/mL at the first sampling time point as a free concentration), suggesting that the active uptake of PA via rOCT2 from the renal blood compartment into the rat kidney is a linear process. To the best of our knowledge, the affinity constants of PA and NAPA with rMATE1 have not been published. However, assuming that there is little species difference between the affinities of various drugs with rMATE1 and hMATE1 [70], the affinity of PA with rMATE1 would appear to be sufficiently large for the assumption that apical efflux of PA into the urine (i.e., *K_m_* value of 1230 μM via hMATE1) [15]. According to the literature [65], the uptake rate of ^14^C-labeled procainamide in HEK293 cells stably expressing rMATE1 was increased up to approximately 1.4 fold of that in HEK293 cells transfected with empty vector. This slight increase in the uptake rate of PA via rMATE1 transporter may be due to the low affinity of PA. Similarly, linear pharmacokinetics were also assumed for the transport kinetics of NAPA in this study.

Regarding the disposition of cimetidine, a minimal PBPK model was applied, since the volume of distribution at steady state for cimetidine was underestimated by the prediction method using the physicochemical properties of cimetidine [50]. The *IC*_50_ value of cimetidine on the metabolic conversion from PA to NAPA was found to be 2060 ± 242 μM. At a cimetidine dose of 100 mg/kg, the effective concentration (i.e., as a free concentration) in the plasma was observed to be below 0.750 mM, suggesting that the inhibition of the conversion of PA to NAPA by cimetidine could be assumed negligible in this study. In the literature, the affinity of cimetidine with rOCT2 and rMATE1 was reported with *K_m_* values of 71.5 μM and 3.01 μM, respectively [63,71]. Thus, we focused on the pharmacokinetics of PA and NAPA affected by cimetidine, but the possibility of drug-drug interactions of cimetidine as a victim drug with PA or NAPA as perpetrators cannot be ruled out since the renal elimination pathway of PA, NAPA, and cimetidine may be shared. Therefore, it may warrant further studies on the possible interactions of the cimetidine pharmacokinetics with PA and NAPA.

## 5. Conclusions

In this study, we proposed a PBPK model that describes the pharmacokinetics of PA and NAPA in the absence or presence of cimetidine. Using our refined PBPK model in this study, the observed drug-drug interactions of PA and NAPA with cimetidine was successfully predicted with respect to the plasma concentration and urinary excretion profiles of PA and NAPA. Our sensitivity analysis for the pharmacokinetics of PA and NAPA suggested that the inhibitory effect of cimetidine via rMATE1 may be important in the renal elimination kinetics of PA and NAPA in rats. The proposed PBPK model describing the pharmacokinetics of PA, NAPA and cimetidine may be a useful tool to understand the mechanism of DDIs between PA/NAPA and cimetidine in vivo.

## Figures and Tables

**Table 1:** Summary of physiological and anatomical variables used for PBPK calculations of PA, NAPA and cimetidine in rats.

**Whole-Body PBPK Model ^a^**		
**Tissue**	**Tissue Volume (mL)**	**Blood Flow (mL/min)**
Adipose	16.7	4.72
Bone	15.7	8.08
Brain	1.24	1.12
Gut	6.19	8.08
Heart	1.05	3.2
Liver	8.57	19.4
Lung	1.24	80
Muscle	116	19
Skin	39.4	4.08
Spleen	0.57	0.88
Venous blood	10.2	-
Arterial blood	5.11	-
**Semi-Mechanistic Kidney ^b^**		
**Parameter**	**Definition**	**Value**
$V_{GLM}$ (mL)	Volume of glomerulus	0.08 [43]
$V_{PTC}$ (mL)	Volume of proximal tubule cells	1.03 [43]
$V_{RBL}$ (mL)	Volume of renal blood	0.375 [43]
$Q_{KI}$ (mL/min)	Blood flow to kidneys	11.6 [42,44]
GFR (mL/min)	Glomerular filtration rate	1.31 [9]
$V_{S1\_1}$ (mL), $Q_{S1\_1}$ (mL/min)	Volume and flow of filtrate in the lumen of 1st sub-segment of S1 segment of proximal tubule	GFR
$V_{S1\_2}$ (mL), $Q_{S1\_2}$ (mL/min)	Volume and flow of filtrate in the lumen of 2nd sub-segment of S1 segment of proximal tubule	0.85 GFR [45,46,47]
$V_{S1\_3}$ (mL), $Q_{S1\_3}$ (mL/min)	Volume and flow of filtrate in the lumen of 3rd sub-segment of S1 segment of proximal tubule	0.70 GFR [45,46,47]
$V_{S2+S3}$ (mL), $Q_{S2+S3}$ (mL/min)	Volume and flow of filtrate in the lumen of S2 and S3 segments of proximal tubule	0.55 GFR [45,46,47]
$V_{LOH}$ (mL), $Q_{LOH}$ (mL/min)	Volume and flow of filtrate in the lumen of Loop of Henle	0.33 GFR [45,46,47]
$V_{DT+CD}$ (mL), $Q_{DT+CD}$ (mL/min)	Volume and flow of filtrate in the lumen of distal tubules and collecting ducts	0.18 GFR [45,46,47]
$V_{U}$ (mL), $Q_{U}$ (mL/min)	Volume and flow of urine	0.02 GFR [45,46,47]

^a^ Parameters (tissue volume and blood flow to tissues) were obtained from the literature [41], which are also used in the Simcyp Software [42] Version 15 Release 1 (Simcyp Limited, Sheffield, UK). ^b^ Semi-mechanistic kidney model [32,33] was incorporated with minor modifications.

**Table 2:** Input parameters for PBPK modeling of PA and NAPA.

Parameter	Value (CV%)		Comments
	PA	NAPA	
**Physchem and Blood Binding**			
Molecular weight	235.33	277.36	-
Compound type	Monoprotic base		-
pKa	9.04	9.04	Predicted ^a^
log P	0.83	0.93	Predicted ^a^
$f_{up}$	0.870	0.688	Measured (PA), estimated (NAPA)
B/P ratio (R)	1	1	Assumed (see text) [28]
**Distribution ($K_{p}$)**			
Kidney	8.31	31.2	Corrected with ER ^b^
Liver	11.7	19.6	Corrected with ER ^b,c^
Brain, heart, lung, and spleen	-	-	Determined (Same with $K_{p,ss}$) ^c^
Other tissues	-	-	Predicted—Rodgers and Rowland method [49]
**Semi-Mechanistic Kidney**			
$CL_{u,int,r}$ (mL/min)	4.67 (16.0)	9.16 (22.5)	Retrograde calculation ^b^
$PS_{in}$ (mL/min)	16.2	20.3	Retrograde calculation ^b^
$PS_{out}$ (mL/min)	7.61	7.61 ^d^	Scaled from PAMPA permeability [44,54] ^e^
$CL_{rabs}$ (mL/min)	0.415	0.415 ^d^	Retrograde calculation ^b^
ER ^f^	0.316	0.327	Determined (see text)
$f_{u,kidney}$	0.223	0.0588	Predicted—Rodgers and Rowland method [49]
**Non-Renal Elimination**			
$CL_{u,int}$ (mL/min)	69.0	2.88	Retrograde calculation ^b^
ER ^f^	0.756	0.0918	Determined (see text)
F_NAPA_	0.534		Retrograde calculation ^b^

^a^ ChemAxon MarvinSketch 15.5.11.0 (http://www.chemaxon.com/products/marvin); ^b^ See text for detailed calculations; ^c^ For the co-treatment condition, $K_{p}$ values for brain, heart, lung, and spleen tissues were adopted from the $K_{p,ss}$ values experimentally determined in this study. For the liver compartment, extraction ratio estimated from calculated CL_NR_ of PA and NAPA was used for the correction of $K_{p,ss}$; ^d^ In this study, parallel artificial membrane permeability assay (PAMPA) permeability and reabsorption clearance of NAPA was assumed to be the same with PA; ^e^ $S_{eff}$ normalized by g kidney was multiplied by the volume of proximal tubule cells, assuming the tissue density is unity; ^f^ Extraction ratio determined for the control group.

**Table 3:** Input parameters for PBPK modeling of cimetidine.

Parameter	Value (CV%)	Comments
**Physchem and Blood Binding**		
Molecular weight	252.34	-
Compound type	Monoprotic base	-
pKa	6.9	Measured [58,59]
log P	0.48	Measured [58]
$f_{up}$	0.836	Measured [56]
B/P ratio (R)	1	Assumed (see text) [57]
**Distribution**		
$V_{C}$ (mL)	83.7 (6.78)	Fitted
$V_{1}$ (mL)	125 (63.4)	Fitted
$V_{2}$ (mL)	200 (80.0)	Fitted
$CL_{D1}$ (mL/min)	17.6 (22.7)	Fitted
$CL_{D2}$ (mL/min)	2.96 (138)	Fitted
$K_{p,KI}$	10.3	Corrected with ER using $K_{p,ss}$ measured [60]
**Semi-Mechanistic Kidney**		
$CL_{u,int,r}$ (mL/min)	0.140	Retrograde calculation ^a^
$PS_{in}$ (mL/min)	839	Retrograde calculation ^a^
$PS_{out}$ (mL/min)	74.1	Scaled from PAMPA permeability [44,61]
ER	0.102	Determined (see text)
$f_{u,kidney}$	0.918	Predicted – Rodgers and Rowland method [49]
**Non-Renal Elimination**		
CL_H_ (mL/min)	2.79	Calculated (see text)
**Transporter Inhibition**		
$K_{i,rOCT2}$ (μM)	9.4	Measured [62]
$K_{i,rMATE1}$ (μM)	3.01	Assumed (see text) [63]

^a^ See text for detailed calculations.

**Table 4:** Observed pharmacokinetic parameters of PA, NAPA (after intravenous administration of 10 mg/kg PA HCl) and cimetidine (after intravenous administration of 100 mg/kg cimetidine) (*n* = 4 for each group).

Parameter	PA		NAPA		Cimetidine
	Control (without Cimetidine)	Co-Treatment (with Cimetidine)	Control (without Cimetidine)	Co-Treatment (with Cimetidine)	
*AUC*_inf_ (µg·min/mL)	140 ± 25.2	223 ± 17.2 **	205 ± 85.9	495 ± 128 **	5220 ± 1410
*t*_1/2β_ (min)	50.4 ± 8.38	38.2 ± 4.70 *	153 ± 15.6	158 ± 38.1	136 ± 77.7
*MRT* (min)	34.3 ± 6.75	31.7 ± 2.36	217 ± 12.3	171 ± 13.5*	80.9 ± 15.0
*V*_SS_ (mL/kg)	2430 ± 539	1520 ± 133 *	-	-	1600 ± 318
*CL* (mL/min/kg)	73.3 ± 11.3	45.1 ± 3.64 **	-	-	20.3 ± 5.68
*CL*_R_ (mL/min/kg)	13.4 ± 2.07	1.88 ± 0.152 **	15.2 ± 4.86	6.51 ± 2.24 *	-
*CL*_NR_ (mL/min/kg)	60.0 ± 9.27	43.3 ± 3.48 *	-	-	-
*C*_max_ (µg/mL)	-	-	0.947 ± 0.292	1.59 ± 0.291 *	-
*T*_max_ (min)	-	-	18.8 ± 7.50	45.0 ± 17.3 *	-
*AUC*_NAPA_/*AUC*_PA_	-	-	1.43 ± 0.331	1.94 ± 0.516	-

* *p* < 0.05 and ** *p* < 0.001 compared with control rats.

**Table 5:** Tissue-to-plasma concentration ratio at steady state ($K_{p,ss}$) of PA and NAPA for 6 major tissues (*n* = 5 for each group).

Tissue	PA		NAPA	
	Control (without Cimetidine)	Co-Treatment (with Cimetidine)	Control (without Cimetidine)	Co-Treatment (with Cimetidine)
Brain	0.200 ± 0.0208	0.246 ± 0.0584	0.713 ± 0.0204	0.0893 ± 0.0710 **
Heart	3.45 ± 1.19	1.80 ± 0.821 *	12.7 ± 2.55	2.40 ± 0.728 **
Kidney	5.68 ± 2.01	9.71 ± 2.30	21.0 ± 5.95	12.5 ± 2.87 *
Liver	2.86 ± 2.11	0.844 ± 0.254	17.8 ± 7.00	6.89 ± 2.68 *
Lung	2.52 ± 1.10	0.547 ± 0.202 **	9.68 ± 2.56	2.65 ± 0.499 **
Spleen	1.34 ± 0.424	1.49 ± 0.590	7.60 ± 1.52	4.69 ± 1.90 *

* *p* < 0.05 and ** *p* < 0.001 compared with control rats.

## Appendix A

For the pharmacokinetics of PA and NAPA, a whole-body physiologically based pharmacokinetic model including eleven major tissues was applied in this study. Based on our preliminary simulations, the perfusion-limited model was considered to be appropriate for the pharmacokinetics of PA and NAPA. Thus, the differential equation for non-eliminating organs (i.e., tissues except for kidney and liver) may be expressed as follows (Equation (A1)):

(A1) $$V_{T}\frac{dC_{T}}{dt}=Q_{T}\cdot \left(C_{art}-\frac{C_{T}\cdot R}{K_{p}}\right)$$

where $V_{T}$ is the volume of tissue compartment; $C_{T}$ and $C_{art}$ are the drug concentrations in the tissue and arterial blood compartments, respectively; $Q_{T}$ is the blood flow to the tissue; R is the blood-to-plasma concentration ratio; $K_{p}$ is the equilibrium tissue-to-plasma concentration ratio.

For liver tissue compartment (Equation (A2)):

(A2) $$\begin{array}{l} V_{LI}\frac{dC_{LI}}{dt}=\left(Q_{LI}-Q_{GU}-Q_{SP}\right)\cdot C_{art}+Q_{GU}\frac{C_{GU}\cdot R}{K_{p,GU}}+Q_{SP}\frac{C_{SP}\cdot R}{K_{p,SP}}-Q_{LI}\frac{C_{LI}\cdot R}{K_{p,LI}}\\ \;\;\;\;\;\;-CL_{u,int}\frac{f_{up}}{K_{p,LI}}C_{LI} \end{array}$$

where $V_{LI}$ is the volume of the liver; $C_{LI}$, $C_{GU}$, and $C_{SP}$ are the drug concentrations in the liver, gut, and spleen, respectively; $Q_{LI}$, $Q_{GU}$, and $Q_{SP}$ are the blood flow to liver, gut, and spleen, respectively; $K_{p,LI}$, $K_{p,GU}$, and $K_{p,SP}$ are the equilibrium tissue-to-plasma concentration ratios for liver, gut, and spleen, respectively; and $CL_{u,int}$ is the intrinsic clearance of drug molecules in the liver compartment.

In the venous blood compartment (i.e., dosing compartment) (Equation (A3)):

(A3) $$\begin{array}{cc} V_{ven}\frac{dC_{ven}}{dt}=Q_{AD} & \frac{C_{AD}\cdot R}{K_{p,AD}}+Q_{BO}\frac{C_{BO}\cdot R}{K_{p,BO}}+Q_{BR}\frac{C_{BR}\cdot R}{K_{p,BR}}+Q_{HE}\frac{C_{HE}\cdot R}{K_{p,HE}}+Q_{LI}\frac{C_{LI}\cdot R}{K_{p,LI}}\\ & +Q_{MU}\frac{C_{MU}\cdot R}{K_{p,MU}}+Q_{SK}\frac{C_{SK}\cdot R}{K_{p,SK}}+(Q_{KI}-Q_{U})\cdot C_{RBL}+Q_{RE}\cdot C_{art}\\ & -Q_{CO}\cdot C_{ven}-Dose\;rate \end{array}$$

where $V_{ven}$ is the volume of venous blood; $C_{AD}$, $C_{BO}$, $C_{BR}$, $C_{HE}$, $C_{MU}$, $C_{SK}$, $C_{RBL}$, and $C_{ven}$ are the drug concentrations in the adipose, bone, brain, heart, muscle, skin, renal blood, and venous blood compartment, respectively; $Q_{AD}$, $Q_{BO}$, $Q_{BR}$, $Q_{HE}$, $Q_{MU}$, $Q_{SK}$, $Q_{KI}$, and $Q_{RE}$ are the blood flows to the adipose, bone, brain, heart, muscle, skin, and kidney and the residual blood flow, respectively; $Q_{U}$ and $Q_{CO}$ are the urinary flow and cardiac output, respectively; and $K_{p,AD}$, $K_{p,BO}$, $K_{p,BR}$, $K_{p,HE}$, $K_{p,MU}$, and $K_{p,SK}$ are the equilibrium tissue-to-plasma concentration ratio of adipose, bone, brain, heart, muscle, and skin, respectively. Dose rate is the dosing rate of drugs to the venous blood.

In the lung compartment (Equation (A4)):

(A4) $$V_{LU}\frac{dC_{LU}}{dt}=Q_{CO}\cdot \left(C_{ven}-\frac{C_{LU}\cdot R}{K_{p,LU}}\right)$$

where $V_{LU}$ is the volume of the lung; $C_{LU}$ is the drug concentration in the lung; $K_{p,LU}$ is the equilibrium tissue-to-plasma concentration ratio for lung.

In the arterial blood compartment (Equation (A5)):

(A5) $$V_{art}\frac{dC_{art}}{dt}=Q_{CO}\cdot \left(\frac{C_{LU}\cdot R}{K_{p,LU}}-C_{art}\right)$$

where $V_{art}$ is the volume of arterial blood.

In the present study, a minimal PBPK model with two tissue compartments was considered to describe the pharmacokinetics of cimetidine. For both peripheral compartments, differential equations could be expressed as follows (Equations (A6) and (A7)):

(A6) $$V_{1}\frac{dC_{1}}{dt}=CL_{D1}\cdot \left(C_{p}-C_{1}\right)$$

(A7) $$V_{2}\frac{dC_{2}}{dt}=CL_{D2}\cdot \left(C_{p}-C_{2}\right)$$

where $V_{1}$ and $V_{2}$ are the apparent volumes of distribution to the two peripheral compartments, respectively; $C_{1}$, $C_{2}$, and $C_{p}$ are the cimetidine concentrations in the first and second tissue compartments, and the plasma, respectively; and $CL_{D1}$ and $CL_{D2}$ are the distributional clearances into and out of both compartments, respectively.

The pharmacokinetics of cimetidine in the central compartment was described using the following equation (Equation (A8)):

(A8) $$V_{C}\frac{dC_{p}}{dt}=CL_{D1}\cdot \left(C_{1}-C_{p}\right)+CL_{D2}\cdot \left(C_{2}-C_{p}\right)-CL_{H}\cdot C_{p}-Q_{KI}\cdot C_{p}+\left(Q_{KI}-Q_{U}\right)\cdot C_{RBL}$$

where $V_{C}$ is the apparent volume of distribution of cimetidine in the central compartment; $CL_{H}$ is the hepatic clearance with regard to the plasma concentration of cimetidine.

In this study, a semi-mechanistic kidney model was incorporated into the final PBPK model to predict drug-drug interactions between PA/NAPA and cimetidine in the process of basolateral uptake and apical efflux. The symbols used for physiological input parameters in the model are summarized in Table 1, and the compound-specific input parameters were obtained from Table 2 and Table 3 for PA, NAPA, and cimetidine: It was noted that the pharmacokinetic variables in the differential equations, especially for semi-mechanistic kidney compartments, are applied for each compound (e.g., $f_{up}$, R, and $K_{p,KI}$). Since drug molecules delivered to rat glomerulus are drained with a filtration rate of $f_{up}GFR/R$, the differential equation for the glomerulus may be expressed as follows (Equation (A9)):

(A9) $$V_{GLM}\frac{dC_{GLM}}{dt}=Q_{KI}\cdot C_{art}-f_{up}GFR/R\cdot C_{GLM}-\left(Q_{KI}-f_{up}GFR/R\right)\cdot C_{GLM}$$

where $C_{GLM}$ is the drug concentration in the glomerulus. Fluid reabsorption from the three S1 segments of proximal tubules was described as follows (Equations (A10)–(A12)):

(A10) $$V_{S1\_1}\frac{dC_{S1\_1}}{dt}=f_{up}GFR/R\cdot C_{GLM}-Q_{S1\_2}\cdot C_{S1\_1}$$

(A11) $$V_{S1\_2}\frac{dC_{S1\_2}}{dt}=Q_{S1\_2}\cdot C_{S1\_1}-Q_{S1\_3}\cdot C_{S1\_2}$$

(A12) $$V_{S1\_3}\frac{dC_{S1\_3}}{dt}=Q_{S1\_3}\cdot C_{S1\_2}-Q_{S2+S3}\cdot C_{S1\_3}$$

where $C_{S1\_1}$, $C_{S1\_2}$, and $C_{S1\_3}$ are the drug concentration in the first, second, and third compartments of the S1 segment of proximal tubules, respectively. In this study, the renal secretion and reabsorption processes were assumed to occur in the S2 and S3 segments of proximal tubules as follows (Equation (A13)):

(A13) $$V_{S2+S3}\frac{dC_{S2+S3}}{dt}=Q_{S2+S3}\cdot C_{S1\_3}-Q_{LOH}\cdot C_{S2+S3}+CL_{u,int,r}\cdot f_{u,kidney}\cdot C_{PTC}-CL_{rabs}\cdot C_{S2+S3}$$

where $C_{S2+S3}$ and $C_{PTC}$ are the drug concentrations in the S2 and S3 segments and proximal tubule cell compartment, respectively; $CL_{u,int,r}$ is the renal intrinsic clearance of drugs from the proximal tubule cell compartment into the S2 and S3 segments; $CL_{rabs}$ is the reabsorption clearance of drugs into the proximal tubule cell compartment; and $f_{u,kidney}$ is the free fraction of drugs in kidney cells (i.e., renal proximal tubule cells).

The remaining fraction of drugs avoiding glomerular filtration was considered to be delivered to a renal blood compartment, in which drug molecules are transported into and out of the proximal tubule cell compartment (Equation (A14)):

(A14) $$V_{RBL}\frac{dC_{RBL}}{dt}=\left(Q_{KI}-f_{up}GFR/R\right)\cdot C_{GLM}-\left(Q_{KI}-Q_{U}\right)\cdot C_{RBL}-f_{up}PS_{in}/R\cdot (C_{RBL}-\frac{C_{PTC}\cdot R}{K_{p,KI}})$$

where $C_{PTC}$ is the drug concentration in the proximal tubule cell compartment, and $K_{p,KI}$ is the equilibrium tissue-to-plasma concentration ratio, which could be also expressed as the following (Equation (A15)):

(A15) $$K_{p,KI}=\frac{f_{up}PS_{in}}{f_{u,kidney}PS_{out}}$$

where $PS_{in}$ and $PS_{out}$ are the tissue permeabilities of drugs into and out of proximal tubule cells, respectively. Essentially, $K_{p,uu}$ can be calculated as the ratio of $PS_{in}$ to $PS_{out}$, whereas $K_{p,KI,pass}$ can be expressed as the ratio of $f_{up}$ to $f_{u,kidney}$ [49,50]. For the proximal tubule cell compartment, the differential equation may be described as follows (Equation (A16)):

(A16) $$\begin{array}{cc} V_{PTC}\frac{dC_{PTC}}{dt}= & f_{up}PS_{in}/R\cdot (C_{RBL}-\frac{C_{PTC}\cdot R}{K_{p,KI}})-CL_{u,int,r}\cdot f_{u,kidney}\cdot C_{PTC}\\ & +CL_{rabs}\cdot C_{S2+S3} \end{array}$$

After two third of fluid was reabsorbed from the proximal tubules, one-third of the remaining fluid enters the Loop-of-Henle, in which 15% of the filtered fluid is reabsorbed, as described in Equation (A17):

(A17) $$V_{LOH}\frac{dC_{LOH}}{dt}=Q_{LOH}\cdot C_{S2+S3}-Q_{DT+CD}\cdot C_{LOH}$$

where $C_{LOH}$ is the drug concentration in the Loop-of-Henle. As described in previous literature [32,33], the kidney model used in this study assumes that the lumen of the distal nephron segments mainly consists of the distal tubules and collecting ducts, which are considered to be kinetically indistinguishable, and receives approximately 18% of the filtered fluid. Since about 16% of fluid reabsorption of the total filtrate was known to occur from this compartment, the urine flow rate ($Q_{U}$) of 2% of the filtration rate was considered as described in Table 1 (Equations (A18)–(A20)) [46,47,48]:

(A18) $$V_{DT+CD}\frac{dC_{DT+CD}}{dt}=Q_{DT+CD}\cdot C_{LOH}-Q_{U}\cdot C_{DT+CD}$$

(A19) $$V_{U}\frac{dC_{U}}{dt}=Q_{U}\cdot C_{DT+CD}-Q_{U}\cdot C_{U}$$

(A20) $$\frac{dA_{E}}{dt}=Q_{U}\cdot C_{U}$$

When PA was co-administered with cimetidine, inhibitory effects were considered in the kinetic process of renal elimination of PA and NAPA. Distribution of PA and NAPA into the proximal tubule cell compartment ($PS_{in}$) without cimetidine might involve active ($PS_{act}$) and passive transport ($PS_{pas}$):

(A21) $$K_{p,KI}=\frac{PS_{act}+PS_{pas}}{PS_{pas}}K_{p,KI,pass}$$

where $PS_{pas}$ may be estimated to be $PS_{out}$, the tissue permeability out of the proximal tubule cell compartment. Since the active transport of PA and NAPA may be inhibited by cimetidine, distribution of PA and NAPA may be expressed as follows:

(A22) $$K_{p,KI}=\frac{\frac{PS_{act}}{1+\frac{{\left[I\right]}_{u,plasma}}{K_{i,rOCT2}}}+PS_{pas}}{PS_{pas}}K_{p,KI,pass}$$

where ${\left[I\right]}_{u,plasma}$ is the free concentration of cimetidine in the plasma (i.e., $f_{up}C_{p}$); $K_{i,rOCT2}$ is the inhibition constant of cimetidine on the active transport of PA or NAPA via rOCT2, assuming that the active process is mainly carried out by the transporter. The efflux kinetics of PA and NAPA when PA was co-administered with cimetidine may be expressed as follows:

(A23) $$CL_{u,int,r,inhibited}=\frac{CL_{u,int,r,control}}{1+\frac{{\left[I\right]}_{u,PTC}}{K_{i,rMATE1}}}$$

where ${\left[I\right]}_{u,PTC}$ is the free concentration of cimetidine in the proximal tubule cell compartment (i.e., $f_{u,kidney}\cdot C_{PTC}$); $K_{i,rMATE1}$ is the inhibition constant of cimetidine on the efflux clearance of PA or NAPA via rMATE1, on the basis of the assumption that the efflux transport is mainly mediated by the transporter.
